# ChatGPT as a Tool for Perioperative Safety: Evaluating Drug Interaction Detection in Anesthetic Regimens

**DOI:** 10.7759/cureus.104836

**Published:** 2026-03-07

**Authors:** Da Young Lee, Sarah Simon, Rose Berkun, Noël C Barengo

**Affiliations:** 1 Medicine, Herbert Wertheim College of Medicine, Florida International University, Miami, USA; 2 Anesthesiology, Herbert Wertheim College of Medicine, Florida International University, Miami, USA; 3 Anesthesiology, Jacobs School of Medicine and Biomedical Sciences, University of Buffalo, New York, USA; 4 Medical and Population Health Sciences, Education, and Research, Herbert Wertheim College of Medicine, Florida International University, Miami, USA

**Keywords:** anesthetic regimens, artificial intelligence, chatgpt, clinical decision support, drug-drug interactions, drug safety, large language models, medication safety, perioperative care, polypharmacy

## Abstract

Background

Drug-drug interactions (DDIs) are a major contributor to perioperative complications, yet no prior studies have evaluated the performance of large language models in this high-risk setting. This study assessed the accuracy of ChatGPT, GPT-5, free version (OpenAI, San Francisco, USA) in identifying DDIs in anesthetic regimens compared with the gold-standard reference Lexicomp.

Methods

We conducted an analytical cross-sectional study using 40 synthetic perioperative vignettes generated by OpenEvidence and reviewed by an anesthesiologist. Each vignette was reviewed for clinically significant DDIs using Lexicomp’s “Interactions” feature. ChatGPT was queried twice with standardized prompts, and responses were classified as Correct or Incorrect.

Results

Across both trials, ChatGPT accurately identified 76 of 80 clinically significant DDIs (sensitivity 95%) with four responses classified as Incorrect. These responses were not entirely erroneous, as each recognized at least one major interaction, yet they omitted additional clinically relevant interactions or failed to provide comprehensive management recommendations. Notably, ChatGPT frequently supplemented its responses with relevant contextual information, including age-specific considerations and perioperative monitoring strategies.

Conclusion

ChatGPT demonstrated high accuracy in detecting clinically significant DDIs in perioperative anesthetic regimens. Its contextual insights may enhance clinical decision-making; however, occasional omissions and limitations in sourcing warrant cautious integration into practice. Further studies in real-world perioperative settings are needed.

## Introduction

Medication safety is a cornerstone of perioperative care, where rapid initiation and modification of anesthetic drug regimens demand accurate, real-time clinical judgment. Drug-drug interactions (DDIs) are a well-established contributor to perioperative complications, ranging from delayed emergence to hemodynamic instability and, in severe cases, life-threatening events. These risks are heightened in patients with polypharmacy or complex comorbidities, making DDI recognition a critical competency for anesthesiologists and perioperative teams [1].

As artificial intelligence (AI) tools become increasingly integrated into healthcare decision-making, large language models (LLMs) such as ChatGPT (OpenAI, San Francisco, USA) have emerged as novel tools with potential applications in clinical support. While initially popularized for general-purpose text generation, ChatGPT has demonstrated utility in various healthcare domains, including patient education, differential diagnosis, and medical documentation [2-5]. More recently, attention has turned to its possible role in pharmacovigilance, specifically identifying DDIs in clinical contexts. Preliminary research has shown that ChatGPT can often recognize and explain basic DDIs, but its reliability varies across clinical settings, drug classes, and case complexity [2]. Some studies have reported promising results in medication therapy management and patient-facing inquiries [3,4], while others note significant limitations in specificity, contextual reasoning, and consistency compared to standard reference tools such as Lexicomp or Micromedex [5,6]. 

Several studies illustrate this evolving landscape. Roosan et al. evaluated ChatGPT-4.0 in medication therapy management across cases of varying complexity and found that it correctly identified DDIs and proposed appropriate management plans in 100% of 39 cases, although dosing recommendations were not provided. Limitations included a small sample size, lack of real patient data, and no assessment of clinical utility or user trust [3]. In contrast, Krishnan et al. assessed ChatGPT-3.5 using hospitalized patient data and reported low sensitivity (0.064-0.16) despite high specificity (>95%), with poor agreement with pharmacist assessments (Cohen’s kappa 0.077-0.143). Approximately 75% of interactions were missed, underscoring the need for specialized datasets and advanced AI models before clinical implementation [7]. Similarly, Juhi et al. found that ChatGPT accurately identified 39 of 40 common DDIs, but only 47.5% of explanations were deemed conclusive, with responses written at a high reading level, limiting accessibility [8]. Thapa et al. further demonstrated that ChatGPT-4.0 correctly identified potential DDIs in discharge prescriptions but was less accurate in characterizing severity, onset, and documentation quality, highlighting the necessity of professional oversight [9].

Together, these studies underscore ChatGPT’s emerging role as a supplementary tool for DDI detection. Its strengths include general identification and explanation of common interactions, particularly in low-risk or educational settings. However, limitations such as variability across prompts, incomplete recognition of interaction severity or pharmacologic mechanisms, and complex language use remain barriers to deployment in high-stakes clinical environments. Notably, existing research has not evaluated ChatGPT’s performance within perioperative anesthetic regimens, where precise pharmacologic coordination is crucial due to the rapid and high-risk nature of anesthetic care.

Our study addressed this gap by assessing ChatGPT’s accuracy in detecting clinically significant DDIs in anesthetic scenarios, comparing its output against the gold-standard reference Lexicomp. This work contributes to ongoing efforts to safely and effectively integrate LLMs into clinical practice to improve perioperative medication safety.

## Materials and methods

We conducted an analytical cross-sectional study to evaluate the performance of ChatGPT (GPT-5, free version) in identifying clinically significant DDIs in perioperative anesthetic regimens. A set of 40 synthetic medication-vignette pairs, created by OpenEvidence and reviewed and validated by an anesthesiologist to ensure clinical relevance and accuracy. The vignettes represented common perioperative drug combinations in patients with polypharmacy, with a mean age of 71.8 ± 14.6 years (range 28-85).

Clinically significant DDIs were defined based on a combination of severity, potential clinical outcomes, and actionability, using Lexicomp’s interaction categories as a reference. Interactions categorized as Category C (monitor therapy), D (consider therapy modification), or X (avoid combination) were considered clinically significant, while Categories A and B were not. This framework provided an objective, reproducible approach for classifying DDIs.

Each vignette was evaluated using Lexicomp’s interaction tool, which served as the benchmark for comparison. For each interaction, Lexicomp provides associated clinical risks, recommended management strategies, and supporting references.

ChatGPT evaluation

ChatGPT’s responses were recorded and evaluated using a structured rubric designed to assess both the accuracy of DDI identification and the relevance of perioperative recommendations. Responses were classified as Correct if they fully identified the clinically relevant DDI and provided appropriate perioperative guidance, or Incorrect if they failed to identify all DDIs or provided inaccurate information. The rubric was developed specifically for this study based on Lexicomp-defined interaction severity and management recommendations, providing an objective, reproducible scoring framework. While the rubric was not formally validated in prior studies, it was reviewed and refined by an anesthesiologist to ensure clinical applicability and consistency.

Two trials were conducted, with each vignette prompted using a standardized template:

“A [age]-year-old patient on [medication(s)] for [indication] is scheduled for [surgery/procedure] with [anesthesia/analgesic agents] use. Are there any clinically relevant drug interactions or perioperative concerns? Please organize the information by severity level, reliability ratings, and recommended patient management.”

All prompts were identical across both trials and input through two new ChatGPT accounts to minimize bias from prior interactions. The prompts were intentionally minimal in patient-level modifiers, focusing primarily on age, medications, and procedure type, to evaluate ChatGPT’s ability to screen for clinically significant DDIs in a standardized perioperative context. Factors such as comorbidities, chronic medications, allergies, and procedure urgency were not included, as these vignettes were designed for initial screening rather than comprehensive perioperative management. We acknowledge that the absence of these modifiers limits the generalizability of the findings to real-world perioperative decision-making, where patient-specific factors significantly influence DDI relevance and management.

Outputs from both trials were compared against Lexicomp to assess accuracy, completeness, and clinical relevance. Identical prompts produced largely consistent responses across accounts, demonstrating reproducibility within this experimental framework. The minimal-context approach provides a controlled setting to evaluate the baseline capability of ChatGPT in DDI detection, with the recognition that further studies incorporating more patient-level detail would be necessary to fully assess real-world applicability.

Primary outcome

The primary outcome was sensitivity in identifying clinically significant DDIs, defined as the proportion of known DDIs correctly identified by ChatGPT: sensitivity = true positives (TP)/[(TP + false negatives (FN)]. Responses were scored as Correct if all reference DDIs were identified, or Incorrect if one or more reference DDIs were omitted. This binary scoring allowed for a qualitative assessment of completeness and clinical relevance of the model’s perioperative recommendations. As the study included only known DDIs, specificity and false-positive rate were not evaluated.

Statistical analysis

Descriptive statistics summarized ChatGPT response patterns across all vignettes. Sensitivity was calculated by treating Correct responses as true positives and Incorrect responses as false negatives. For Incorrect responses, most errors represented omissions of reference DDIs; in rare cases where ChatGPT suggested additional non-reference interactions, these were documented separately but not counted against sensitivity. Additional qualitative evaluation assessed the clinical appropriateness and completeness of any perioperative recommendations provided by ChatGPT.

## Results

A total of 40 synthetic perioperative vignettes were evaluated using ChatGPT (GPT-5, free subscription). The mean ± SD age of patients was 71.8 ± 14.6 years (range 28-85), with most cases involving elderly individuals undergoing common surgical procedures. Medications assessed predominantly included antidepressants, opioids, antihypertensives, anticoagulants, neuromuscular blockers, and common anesthetics/CNS depressants. Table 1 presents 20 representative cases. 

**Table 1:** Summary of 20 representative synthetic perioperative vignettes, including patient age, sex, surgical procedure, and medications assessed for potential drug-drug interactions. Note: Sex and other demographic variables are descriptive only and were not included in the ChatGPT prompts. ORIF: open reduction and internal fixation.

Case	Age	Sex	Surgical Procedure	Medications
1	78	F	Hip arthroplasty	Sertraline; fentanyl
2	65	M	Elective hernia repair	Amitriptyline; glycopyrrolate
3	82	F	Femoral Fracture ORIF	Warfarin; citalopram; propofol
4	70	M	Prostatectomy	Tramadol; propofol
5	25	F	Coronary artery bypass grafting	Mirtazapine; metoprolol; remimazolam
6	68	M	Colectomy	Paroxetine; oxycodone
7	80	F	Cholecystectomy	Escitalopram; omeprazole; remimazolam
8	77	M	Bowel resection	Venlafaxine; linezolid; propofol
9	69	F	Breast surgery	Fluvoxamine; midazolam
10	85	M	Carotid endarectomy	Sertraline; clopidogrel
11	72	F	Total knee replacement	Duloxetine; morphine
12	74	M	Transurethral resection of prostate	Citalopram; ondansetron
13	67	F	Thyroidectomy	Nortriptyline; sevoflurane
14	79	M	Spinal decompression	Sertraline; ibuprofen
15	73	M	Vascular bypass surgery	Fluoxetine; methadone
16	76	F	Mastectomy	Paroxetine; codeine
17	70	F	Total knee arthroplasty	Sertraline; enoxaparin
18	83	M	Hip fracture repair	Mirtazapine; haloperidol
19	78	M	Craniotomy	Fluoxetine; phenytoin
20	74	M	Coronary artery bypass grafting	Amiodarone; fentanyl

Trial one

Conducted on October 5, 2025, using a Windows PC with Microsoft Edge (Microsoft Corporation, Redmond, USA), ChatGPT correctly identified clinically significant DDIs and provided appropriate perioperative management recommendations in 39 of 40 cases (97.5%). One vignette (Case seven: escitalopram + omeprazole) was graded as Incorrect due to omission of detailed patient management recommendations in Lexicomp, such as alternative acid-suppressive therapy or dose adjustment.

Trial two

Conducted later the same day on an Apple MacBook (Apple Inc., Cupertino, USA) with Chrome browser, ChatGPT correctly identified DDIs in 37 of 40 cases (92.5%). The three incorrect responses included Case three (warfarin + citalopram + propofol), in which propofol’s potential QT-prolonging effect was omitted; Case 11 (duloxetine + morphine), where the additive hypotensive effect was not recognized; and Case 15 (fluoxetine + methadone), in which ChatGPT did not mention fluoxetine’s effect on methadone metabolism. No “negative control” vignettes (with no interactions) were included, as the study focused on assessing the detection of known clinically significant DDIs. Overall, ChatGPT demonstrated high sensitivity in detecting clinically relevant perioperative DDIs, with occasional omissions reflecting minor limitations in clinical detail.

Comparison to Lexicomp

Overall, ChatGPT demonstrated high performance in detecting clinically significant DDIs compared with Lexicomp. Across both trials, the model consistently identified major interactions and provided clinically meaningful perioperative context, aligning closely with Lexicomp recommendations while occasionally offering additional pharmacologic considerations.

Notably, ChatGPT frequently supplemented its responses with additional considerations not explicitly listed in Lexicomp’s “Interactions” tab. For example, in case four (tramadol + propofol), while Lexicomp recommended avoiding the combination if possible, ChatGPT contextualized its response by suggesting careful titration and monitoring when avoidance was not feasible. The model also incorporated age-specific adverse effect concerns, such as increased delirium or fall risk in elderly patients, reflecting its ability to integrate broader pharmacologic context.

Summary statistics

Across both trials, ChatGPT correctly identified 76 of 80 known clinically significant DDIs, yielding a sensitivity of 95% (95% CI, 87.69-98.62%). The denominator of 80 was derived by counting all interactions categorized in Lexicomp as Category C (monitor therapy), D (consider therapy modification), or X (avoid combination) across the 40 vignettes. Incorrect responses primarily reflected omissions of one or more reference DDIs. A per-vignette breakdown of identified interactions and missed DDIs is provided in Table 1. 

## Discussion

Our study demonstrates that ChatGPT (GPT-5, free version) can identify clinically significant DDIs in perioperative anesthetic regimens, achieving an overall identification rate of 95% across 40 synthetic vignettes. Sensitivity was 95%, with occasional partial omissions in clinical detail, such as propofol’s QT-prolonging effects or fluoxetine’s impact on methadone metabolism. These results suggest that ChatGPT may serve as a supplementary tool for screening perioperative medication regimens, providing both accurate DDI detection and clinically relevant context, while professional oversight remains essential.

Our results build on prior work evaluating earlier versions of ChatGPT in medication management [2-9]. Compared with ChatGPT-3.5 and 4.0, GPT-5 achieved higher sensitivity and more consistent identification of clinically relevant interactions within perioperative anesthetic regimens. While previous studies noted omissions in dosing, interaction severity, or explanatory depth, our findings indicate that GPT-5 not only reliably detects DDIs but also provides supplementary clinical considerations, such as age-specific adverse effects and additive pharmacologic risks.

While the model frequently highlighted additive cardiovascular or respiratory depressant effects when combining anesthetics and sedatives, these observations are descriptive of its outputs rather than directly attributable to its training on biomedical literature. Occasional omissions underscore the continued importance of verifying AI-generated guidance against validated references and professional judgment.

This study has several limitations. The use of 40 synthetic vignettes, while standardized, represents a small sample size and may not fully capture the complexity of real-world perioperative care. Only one reference standard (Lexicomp) was used, which may limit generalizability. Additionally, the absence of negative-control vignettes prevented assessment of specificity and false-positive rates. These factors should be considered when interpreting the reported sensitivity and generalizability of findings.

In conclusion, this study provides the first systematic evaluation of ChatGPT (GPT-5, free version) for perioperative anesthetic DDI detection. GPT-5 demonstrates high sensitivity and the ability to offer supplemental contextual insights, supporting its potential role as an adjunct to validated clinical resources. Future research should assess ChatGPT in real-world clinical settings, include negative-control cases, and explore human-AI collaboration in perioperative workflows to optimize safety and efficiency.

## Conclusions

ChatGPT (GPT-5) demonstrated high accuracy in detecting clinically significant DDIs in perioperative anesthetic regimens, with sensitivity approaching that of established reference tools such as Lexicomp. Its ability to provide supplemental contextual insights, including patient-specific considerations and perioperative guidance, may enhance clinical decision-making. However, occasional omissions and the need for confirmatory reference checks highlight that AI tools should be used as an adjunct, not a replacement, for validated clinical resources and professional judgment. Integrating LLMs like ChatGPT into perioperative workflows holds potential to support anesthesiologists, improve medication safety, and streamline pharmacologic review, provided that oversight and adherence to clinical standards are maintained.
